# Tobacco industry corporate social responsibility activities and other interference after ratification of a strong tobacco law in Ethiopia

**DOI:** 10.1136/tc-2023-058079

**Published:** 2023-09-15

**Authors:** Sisay Derso Mengesha, Claire Brolan, Coral E Gartner

**Affiliations:** 1 Environmental Health and Noninfectious Disease, Ethiopian Public Health Institute, Addis Ababa, Ethiopia; 2 School of Public Health, University of Queensland, Herston, Queensland, Australia

**Keywords:** Tobacco industry, Public policy, Social marketing

## Abstract

**Background:**

Since strict new tobacco control laws were introduced in 2019 and 2020, the National Tobacco Enterprise (NTE), the main manufacturer and distributor of cigarettes in Ethiopia, strategically engaged in corporate social responsibility (CSR) activities and other tactics to interfere in tobacco control policymaking. This study systematically identified and reviewed tobacco industry activities that undermine Ethiopia’s strict new tobacco control laws.

**Methods:**

We collated, reviewed and analysed evidence on tobacco industry CSR activities from February 2019 to November 2022 in Ethiopia, including newspapers, organisational websites, social media and government documents related to tobacco industry activities, contract agreements and other policy interference attempts.

**Results:**

NTE’s CSR activities included: (1) Funding educational programmes (eg, postgraduate scholarships); (2) Community service (eg, donating COVID-19 prevention materials, providing water, sanitation and hygiene supplies); and (3) Supporting government programmes (eg, greening initiatives and training programmes). NTE facilitated CSR activities via a contract agreement with the Ethiopian government that was created when Japan Tobacco International purchased the Government’s majority share in NTE. NTE subsequently partnered with the Japanese Embassy in Addis Ababa and private law firms on CSR activities. The tobacco control community stopped NTE distributing free COVID-19 prevention products in Addis Ababa but had limited impact on other identified breaches of laws prohibiting tobacco advertising promotion and sponsorship.

**Conclusion:**

The new laws have not stopped NTE using multiple CSR activities to interfere in tobacco control policy. Regular monitoring of tobacco industry CSR activities to identify potential breaches is recommended. Moreover, the Ethiopian government should revise its contractual agreement with NTE to end NTE’s participation in law-making processes and partnerships on illicit tobacco control.

**WHAT IS ALREADY KNOWN ON THIS TOPIC**

- It is well documented that in all global regions, the tobacco industry (TI) interferes in public health policy for financial gain and expansion of their commercial activities.
- One major way the TI seeks to favourably influence a country’s public health policy and regulatory decision-makers is through corporate social responsibility (CSR) tactics and initiatives.
- During the height of the COVID-19 pandemic crisis, TI interference through CSR activities to support both public and private public health emergency responses occurred in many countries.
- There has been limited research on TI interference in Ethiopia, and no published evaluation of CSR activities by the TI after tobacco control law was enacted in February 2019 to ban such activities.

**WHAT THIS STUDY ADDS**

- This is the first study to review publicly available information and government internal documents that were exchanged with the TI to identify and describe the types of CSR activities and mode of interference in public health policymaking and regulatory initiatives after TI CSR activities were banned in Ethiopia in February 2019.
- The study identified key TI front groups and partners, and strategies that were used by the TI to present itself as a ‘good corporate citizen’ and to engage in unnecessary interaction with the government.
- Our findings provide important knowledge of the profile of TI front groups such as law firms and tobacco farmers.
- The study findings provide new insights for policymakers to protect public health laws from the activities of the TI.

**HOW THIS STUDY MIGHT AFFECT RESEARCH, PRACTICE OR POLICY**

- Our results indicated that CSR activities were used by the TI to gain influence, meddle in life-saving health policies and secure preferential treatment from government organisations.
- Such evidence calls for increased protection of public health laws from financial interests posed by the TI not only in Ethiopia but across Africa and globally.

## Introduction

 Ethiopia’s National Tobacco Enterprise (NTE) was established in 1942 by Proclamation No. 30/1942 as Ethiopia’s tobacco monopoly organisation.^1^ NTE remains the only tobacco company that operates, manufactures, imports and distributes tobacco products in and across the country. In 2017, the Ethiopian government privatised its NTE share to Japan Tobacco International (JTI) for US$1 billion, the most significant purchase in Ethiopia’s privatisation history. Today, NTE is owned by two entities: JTI (71% share) and Yemen-based Sheba Company (29% share).^2^ JTI will control NTE’s monopoly rights until 2025 with an option to renew this agreement in the contract. On the one hand, the Ethiopian government’s withdrawal from NTE demonstrates its commitment to the WHO Framework Convention on Tobacco Control (WHO FCTC) following government ratification of that Convention in January 2014.^3^ However, financial challenges and a domestic uprising in some parts of the country may also have motivated the decision to sell NTE.^4^

Article 5.3 of the WHO FCTC states ‘In setting and implementing their public health policies with respect to tobacco control, Parties shall act to protect these policies from commercial and other vested interests of the tobacco industry in accordance with national law’. Article 5.3 empowers governments to protect themselves from tobacco industry (TI) tactics, such as the use of corporate social responsibility (CSR) activities.^5^ Tobacco companies have historically used CSR to not only remediate their public image, but also to gain access to policymakers and thereby influence public health policy.^6^ The Ethiopian government passed a series of tobacco control laws in 2019 and 2020 (Proclamation No. 1112/2019 and Excise Tax Proclamation No. 1186/2020) in line with its WHO FCTC obligations that included a total ban on TI CSR activities, such as prohibiting individuals with TI connections from participating in tobacco control training, and prohibiting government organisations from receiving any financial or in-kind support from the TI, along with smoke-free public places, graphic pack health warnings, a total ban on tobacco advertising, promotion and sponsorship (including at point of sale), and prohibition of sale to and by minors under 21 years of age.^7^,^9^

Importantly, Proclamation No. 112/2019 prohibits TI interference in government activities by stating that interaction between the TI and health policymakers ‘shall be limited to only those strictly necessary for effective regulation of the tobacco industry or tobacco products’. Proclamation No. 1112/2019 further states that ‘any person who prepares, publishes, transmits, or participates in the illegal or unauthorised advertisement or promotion of tobacco products should be punished by imprisonment not exceeding three years and/or with a fine not less than fifty thousand Birr (~USD$900)’.^7^ In terms of Excise Tax Proclamation No. 1186/2020 introduced in February 2020, Ethiopia’s new tobacco excise law aims to achieve a mixed-excise system for cigarettes consistent with WHO recommendations. The reform applied a 30% tax rate on the retail price of cigarettes and a specific excise rate of Ethiopian Birr (ETB) 8 on each pack (or US$0.25 at February 2020’s exchange rate). The Ministry of Finance is authorised to increase the tobacco tax by 10% each year without seeking authorisation from Ethiopia’s Council of Ministers.^10^

Despite these strong legal measures by the Ethiopian government, the threat of TI interference remains and the 2020 and 2021 Global Tobacco Industry Interference Index Reports for Ethiopia highlight several ways the TI has interfered in policymaking in Ethiopia.^11^,^13^ TI interference most often occurs where there is limited understanding of tobacco control provisions and engagement in tobacco control activities beyond health agencies, and limited authority for coordination mechanisms among regional state and national governments.^14^,^16^ Conflicts of interest at personal and institutional levels among health and non-health agencies can also be barriers to minimising industry interference.^17^ The TI further obstructs government-led tobacco control efforts through various CSR-related tactics and strategies. For instance, to obstruct the passage of Proclamations No. 1112/2019 and No. 1186/2020, Ethiopia’s TI promoted its ‘good’ corporate citizenry as a significant taxpayer and employer.^13^ Indeed, when the Proclamations were in draft form and under public discussion, NTE unsuccessfully used tobacco growers and members of the Addis Ababa Chamber of Commerce to form antitobacco control law alliances at public hearings.^18^ Such protobacco lobbyists attempted to rebuild NTE’s reputation as a platform for voluntary regulation and as a source of trustworthy information.^6^

The TI’s strategic use of CSR activities to interfere in tobacco-control laws in Ethiopia is consistent with Big Tobacco’s leveraging of CSR initiatives globally.^19^ For example, the African Tobacco Control Alliance describes several TI CSR activities across African countries, including a poverty alleviation programme in Tanzania, the construction of a community drinking water facility in Burkina Faso, US$1.2 million in funding to address child labour and strengthen children’s rights in Mozambique, and financial support for a local rugby team in South Africa (among other activities).^20^ Further studies have also described TI CSR activity in low-income and middle-income countries in the Asian region.^21^ However, how Ethiopia’s TI has leveraged CSR narratives since Proclamations No. 1112/2019 and No. 1186/2020 were implemented has not been systematically examined. Therefore, this study aims to identify and document TI CSR activities in contravention of Ethiopia’s new tobacco control laws. To systematically explore this topic in the Ethiopian context postlaw implementation, we systematically reviewed publicly available evidence to identify TI CSR activities after 2019 to document TI image-building and self-promotion, as well as TI interaction with government bodies. As no publicly accessible repository exists mapping TI interference in Ethiopia’s tobacco-control policymaking (in contravention to Ethiopia’s new tobacco control laws), our findings may advance understanding and countering of TI interference in tobacco control policymaking processes not only in Ethiopia but elsewhere.

## Methods

We aimed to identify and review any CSR activities by the TI in Ethiopia since 2019 in contravention of Article 51 of the Ethiopian Food and Drug Authority (EFDA) Proclamation No. 112/2019. We searched online databases using the Google search engine and social media platforms such as Twitter, as well as Telegram for tobacco control-related media stories (Ethiopian and African newspaper websites) for occurrences of TI CSR-related interaction with Ethiopian government agencies and representatives. Key search terms included ‘Ethiopia’, ‘Tobacco Control’, ‘Framework Convention on Tobacco Control’, ‘FCTC’, ‘World Health Organization Framework Contention on Tobacco Control’, ‘WHO FCTC implementation’, ‘Tobacco Industry’, ‘industry interference’, ‘JTI’ and ‘National Tobacco Enterprise’. The search terms were customised for each data source to retrieve published articles and policy documents in Ethiopia from February 2019 until November 2022. Documents were included if they described any TI CSR activity in Ethiopia since the adoption of Proclamation No. 1112/2019 (February 2019). The search of newspaper articles, social media posts and government internal reports from all sources produced 47 results, of which 31 remained after duplicates were removed and these were included in the full-text review (see online supplemental table 1).

To strengthen the search, key public institutions that interact with NTE or have tobacco control regulatory functions were contacted by letter, email and telephone. These were the EFDA (tobacco regulatory body) and the Customs Commission (who signed a memorandum of understanding (MOU) with NTE to fight contraband cigarettes). We requested any correspondence or documents exchanged between these government agencies with the TI that are on the public record but not available online. In addition, we approached a leading tobacco control advocacy civil society organisation (CSO), Mathiwos Wondu Ye-Ethiopia Cancer Society (MWECS) as a key informant on TI CSR-related activities and tobacco control community responses for the CSR activities that we identified in the data. We asked MWECS to provide details of TI CSR activities within the time period of study interest. Overall, 11 documents were obtained from the three organisations, including the NTE privatisation contract agreement document, NTE letters from the Prime Minister’s Office, minute records between EFDA and NTE, email exchanges between TI and EFDA, a letter to the Japanese Embassy in Addis Ababa from CSOs, and others.

Relevant information identified from the on-line and off-line searches, as well as contextual discussions with MWECS, were subsequently entered into tables, with the first author (SDM) leading the thematic analysis of the qualitative data guided by Braun and Clarke’s original framework for thematic analysis.^22^ The thematic analysis was an iterative process, involving SDM becoming familiar with the data by reading and re-reading the material, as well as developing and revising the thematic codes.

## Results

Three main themes were identified in the data:

1. TI’s CSR initiatives since the passing of Proclamations No. 1112/2019 and No. 1186/2020.
2. Questionable interaction between the TI and official government agencies.
3. Countermeasures against TI interference.

### Theme 1: TI CSR activities since the passing of proclamations No. 1112/2019 and No. 1186/2020

NTE, via its major shareholder, JTI, has engaged in CSR activities including providing university scholarships for Ethiopian students and community water supply and sanitation services (table 1). For instance, the Japanese Embassy in Addis Ababa and JTI entered into a MOU on 21 May 2021 to collaboratively provide postgraduate scholarships to Ethiopian students to study in Japan (online supplemental table 1).^23^,^26^ The MOU was signed by the Japanese Ambassador and a JTI representative and promoted as building a mutually beneficial bridge between Japan and Ethiopia. JTI representatives committed to ongoing financial contributions to the scholarship programme.^26^ Moreover, JTI announced via Twitter CSR activities related to providing safe water and improved sanitation services for Ethiopian households, without providing details of the location/communities supplied.^27^

**Table 1:** List of CSR activities carried out by National Tobacco Enterprise (NTE) since the passage of Proclamation No. 1112/2019

Type of CSR-related support from NTE	Partner	Engagement period
Funding scholarships for postgraduate Ethiopian students to study in Japan	Japanese Embassy in Addis Ababa	2021
Providing safe water supplies and toilets (WASH) to more than 3000 households in Ethiopia	Undisclosed	2021
Providing training to Customs Commission staff, including policy officers, on combatting the illicit tobacco trade	Customs Commission	2021
In response to the COVID-19 pandemic, producing and freely distributing hand sanitisers to disadvantaged communities in Lideta, a suburb of Addis Abba where the NTE Head Office is located	Local government of Lideta in Addis Ababa	2020
Supporting Ethiopian tobacco farmers with free seeds and agrochemicals	Smallholder farmers	2020
Publicly participating in tree planting initiatives as part of the Ethiopian government’s Green Legacy national project	Motor & Engineering Company of Ethiopia Limited S.C. (MOENCO), Coca Cola and other five client companies of Mehrteab Leul & Associates Law Office (MLA)	2019

CSRcorporate social responsibility

NTE capitalised on the COVID-19 pandemic in Ethiopia, which commenced in March 2020 when the first case was reported in Addis Ababa.^28^ NTE produced hand sanitiser and distributed it without charge to disadvantaged groups in Lideta subcity in Addis Ababa.^29^ NTE’s head office and factories were located in Lideta at that time, which was among the subcities with the highest recorded numbers of confirmed positive COVID-19 cases.^30^

We identified TI CSR activities connected to the Ethiopian Government’s National Greening Initiative. In July 2019, Ethiopia’s prime minister launched a nationwide four billion tree planting project at Adwa Park in Addis Ababa. At the launch, the prime minister was accompanied by NTE employees who planted over 2000 trees. As part of the National Greening Initiative, NTE further participated in tree planting as part of the country’s national reforestation campaign with seven corporate clients of the Mehrteab Leul & Associates Law Office. This law firm reported that its clients see the initiative as a ‘good opportunity’ to discharge their CSR responsibilities.^31^ On 26 July 2019, Ethiopian Television, the national television broadcaster, broadcasted and promoted the law firm initiative and NTE partnership.^32^ During the press conference, NTE’s corporate affairs and communication director emphasised that the company would plant over 2000 seedlings on Ethiopia’s Green Legacy Day (29 July 2019) and would support the government’s green policies by discharging its CSR obligations.^32 33^ Overall, NTE reported nearly 4000 saplings as part of the Green Legacy programme.

Additionally, we found that Ethiopia’s TI invests in training government workers, including Customs Commission staff, and members of the police and army, on controlling illicit tobacco trade. Email communication between NTE and a branch of the Customs Commission, following an NTE Customs Commission site visit, indicates this training is based on an active, inplace agreement. Three days of training were scheduled at the government Customs Commission branch office in Dire Dawa following the visit.^33^

In June 2020, NTE members published an article about NTE contributions to the ‘Tobacco Production Practice of Smallholders’ on tobacco farms in Bilate, Wolaita and Hawassa.^34^ The article highlighted how NTE supports these smallholder farmers by providing free seeds and pesticides, implicitly as part of NTE’s CSR commitments. The article also suggests that policymakers should foster and facilitate tobacco production and marketing.

### Theme 2: Questionable TI interactions with the government to obtain benefits

Our study found that when JTI bought 71% of the government’s NTE share in two instalments in 2016 (40%) and 2017 (30.9%), JTI received privileges in the sale contract agreement with the government, which enabled the company to interfere in tobacco control efforts. The significant benefits to NTE included participating in law-making processes, obtaining foreign currency for investments, partnering with a government body that controls illicit tobacco and retention of NTE monopoly rights until 2025 (box 1).

Box 1 **Key loopholes in the contractual agreement in the 71% share sale by the Ethiopian government to Japan Tobacco International (JTI).**

1. Conclude a MOU with the NTE, with the objective of reducing the illegal trade of tobacco products, which is significant in Ethiopia. In this regard, the Government’s anti-illegal trade task force and the NTE should collaborate closely to define an action plan, and a list of measures to best ensure such an objective can be achieved.
2. During the law-making process, consulting and participating with relevant stakeholders and the public is a necessary precondition in Ethiopia. To this end, all branches of government and administrations, whose mission and efforts may be directly or indirectly impacted by any proposed modification or preparation of tobacco taxation, regulation or legislation should be consulted. The NTE should be consulted in a transparent manner during modification or preparation of tobacco taxation, regulations or legislation.
3. Take all measures necessary to ensure that the NTE retains its monopoly rights until December 2025, as per the contract of sale signed on 15 July 2016, and Proclamation No.181/1999 to issue business licenses in respect of the purchase, preparation, manufacture, sale, import, and export of tobacco and tobacco products.
4. Facilitate as much as possible the availability of foreign currency as may be reasonably required by the NTE, to implement its business plan.

MOU, memorandum of understanding; NTE, National Tobacco Enterprise.

These examples demonstrate how the TI uses contractual privileges to influence public policy development and to hinder the impact of tobacco control laws. For example, using a contractual loophole, NTE signed an MOU with the Ethiopian Customs Commission to assist in activities to combat the illicit tobacco trade, particularly in eastern Ethiopia.^35^ As in other countries, the TI in Ethiopia has cited inflated figures of the market share of the illicit tobacco trade.^36^ NTE subsequently interfered in excise tax reform, using its MOU with the government to weaken tax provisions, claiming illicit trade accounted for 45% of the market. Consequently, government officials cited this figure during the lawmaking process, giving it credibility. A high-level minister from Ethiopia’s Ministry of Finance is reported to have said, ‘If the country imposes a higher tax, it will push the industry out of the market’.^37^ The TI further used the MOU to advertise its products by labelling NTE tobacco products and its logo on promotional materials as part of combating illicit tobacco products.^38^ Moreover, the MOU allowed the TI to participate as a key stakeholder in government consultations about tobacco tax amendments and other tobacco control directives drafted by the EFDA that became part of Proclamation No. 1112/2019.

TI involvement in lawmaking processes exerts pressure on public health advocacy by delaying legal decision-making and advancing commercial interests by diluting public health provisions. For example, the TI delayed government decision-making by requesting additional time to comment on the proposed tobacco control directive (No. 771/2021)^8^ and an English version of the draft directive.^39^ Nonetheless, the EFDA pushed back against this request:

[The] NTE’s claim that it is mandatory for the Ethiopian Food and Drug Authority to prepare the draft directive in the English language at this early stage of the Directive making process is incorrect. The Federal Administrative Procedure Proclamation No. 1183/2020 provided step-by-step requirements. Relevant to this matter, Proclamation No. 1183/2020 classified the requirements into two parts: (1) “procedures before adoption of a Directive (Articles 7-11), and (2) Ratification and Effectiveness of Directives (Articles 12-19). NTE’s claim that the Ethiopian Food and Drug Authority must prepare the directive in English at this stage is an erroneous understanding of the law. (See online supplemental table 1)

However, during this period, Ethiopia’s Ministry of Revenue awarded NTE a trophy for being a higher and loyal taxpayer for the 2019 fiscal year. The prime minister was present at the award ceremony. Such public recognition of the TI implies government support, endorsement and promotion, which appears to contravene Proclamation No.11121/2019.

### Theme 3: Countermeasures against TI interference through CSR activities

In response to the challenge of TI interference, the EFDA established the TI Monitoring and Response Team as a subcommittee of the National Tobacco Control Coordination Committee. This team uncovered and actively responded to TI CSR activities (table 2). Among NTE’s CSR activities, the production and distribution of COVID-19 prevention hand sanitiser to the residents of Addis Ababa in the Lideta subcity was immediately halted by the EFDA with a countermeasure to stop further production and dissemination of these products. On 6 June 2020, MWECS exposed NTE’s CSR efforts related to COVID-19 prevention as a tactic to regain its acceptability among both the public and the government.^29^ Later in June 2020, MWECS further accused NTE of breaching the ban on advertisement and promotion of tobacco products by using the MOU on combatting Ethiopia’s illicit tobacco trade to promote their products. MWECS exposed NTE’s tactics that included using advertising on wall clocks and plastic bags distributed to shops, bars and restaurants featuring a ‘Stop Contraband’ slogan with NTE’s name displayed underneath.^38^ Unfortunately, there is no evidence of regulatory action against NTE in response to MWECS exposing this advertising.

**Table 2:** Summary of countermeasures against the tobacco industry’s CSR activities in Ethiopia

CSR activity	Countermeasure	Action period	Responsible body	Outcomes
NTE produced hand sanitiser and distributed it in response to the COVID-19 pandemic	EFDA ceased production and distribution	2020	TIMRTEFDAMWECS	NTE stopped from producing and distributing COVID-19 prevention materials
	MWECS publicly exposed the CSR tactic	June 2020	MWECS	Exposed the TI’s presence behind COVID-19 related CSR activities
Providing scholarships to Ethiopian students to study in Japan	CSOs sent a letter expressing concern over Japanese Embassy’s involvement in JTI’s CSR activity	2022	Consortium of Ethiopian NCD AssociationsTIMRTMWECS	No change identified
NTE engagement in Tobacco Advertising Promotion and Sponsorship by using the MOU signed with the Customs Commission	NTE used the MOU agreement with government to promote its products. This activity was exposed by MWECS	2020	MWECS	NTE continues engaging in Tobacco Advertising Promotion and Sponsorship
Recognition of NTE as a loyal taxpayer	The tobacco control community strongly criticised the action of the Ministry of Revenue for recognising NTE as a loyal taxpayer	2020	TIMRTMWECSEFDA	NTE removed from 2020 award list

CSOcivil society organisationCSR, corporate social responsibility; EFDA, Ethiopian Food and Drug Authority; JTI, Japan Tobacco International; MOUmemorandum of understandingMWECS, Mathiwos Wondu Ye-Ethiopia Cancer Society; NCD, non-communicable disease; NTE, National Tobacco Enterprise; TItobacco industryTIMRT, Tobacco Industry Monitoring and Response Team

## Discussion

Our study findings demonstrate the TI’s use of diverse CSR activities to improve its reputation, image and credibility as a corporate citizen in Ethiopia. These activities included funding travel scholarships for students, donating hand sanitiser and personal protective equipment to control COVID-19, and tree planting to reduce climate change. NTE also used CSR activities to promote tobacco products, such as by displaying its corporate trademark and branding on tobacco products during a televised press conference promoting their CSR activities, and on anti-illicit tobacco promotional materials.

Our findings show TI strategic use of public CSR opportunities, such as the Green Legacy Project, to promote an image of itself as socially and environmentally responsible to deflect attention from the health and environmental impacts of tobacco products.^40 41^ These include the negative environmental impacts from tobacco production and curing, product manufacturing and distribution, product consumption, and the waste generated from tobacco use.^42 43^ Tobacco farming uses agrochemicals that reduce soil fertility and soil quality, uses a substantial quantity of water, and contaminates the air and water systems.^44^ Tobacco farming in Africa poses a medium-to-serious degree of tobacco-related deforestation particularly in southern and eastern African countries, with the percentage of tobacco-related deforestation in Ethiopia accounting for close to 1% in 1995.^42 43 45^ Almost 30 years since these estimates were made, it is anticipated this figure is substantially higher. According to the Global Center for Good Governance in Tobacco Control, harmful waste made up of cigarette butts and packs costs Ethiopia ETB911 million per year in water pollution and waste management.^46^ These environmental impacts, in addition to the well-known health impacts of tobacco smoking,^47^ are incompatible with Ethiopia’s Sustainable Development Goal (SDG) commitments, especially to achieve the health targets in SDG 3, notably the specific target of reducing premature mortality from non-communicable diseases by a third by 2030.^48^

Our study highlights how NTE used the COVID-19 pandemic to develop an image of providing health-promoting assistance during a public health crisis through actively leading the distribution of hand sanitiser and personal protective equipment to disadvantaged communities. Similarly, in other global regions such as Europe, multinational tobacco companies used the COVID-19 pandemic to generate media attention to raise their corporate profile, advertise items like Heated Tobacco Products and to normalise tobacco use.^49^ For instance, a tobacco company donated ventilators to the government of Greece.^50^ Elsewhere in the world, the Canadian government partnered with Philip Morris International on COVID-19 vaccine development, providing an opportunity for the company to issue a press release that publicised the partnership.^51^ Such activities that exploit a public health crisis involving a respiratory disease are particularly inappropriate given tobacco is the leading cause of preventable death from respiratory diseases globally.^52^

Tobacco companies have increased spending on CSR activities in countries where governments have adopted comprehensive bans on tobacco advertising, promotion and sponsorship, or severely restricted these marketing opportunities.^53^ Indeed, our results show that NTE has participated in multiple CSR activities since 2019 despite new laws expressly prohibiting this activity.^7^ We also found no evidence that NTE was penalised for these activities. Additional capacity-building and funding may be required to ensure total advertising restrictions, such as those in Ethiopia, are effectively enforced.^54^ In this regard, our study confirms that transnational tobacco companies can exploit CSR initiatives as one of their political operations to interfere with tobacco control efforts and to promote their products in low-income and middle-income countries.^55^

Proclamation No. 1112/2019^7^ Article 51(3) and (4) forbids the Ethiopian government from accepting any assistance from the TI on any enforcement activities or entering into any partnership with the TI. However, in contravention of Article 5.3 of the WHO FCTC, our findings confirm NTE receives strong government support via crucial articles in the contract agreement that transferred the government’s NTE shares to JTI in 2017 (box 1), predating the country’s new tobacco control law passed in 2019. The relationship between JTI and the Ethiopian government through NTE ownership has wider implications for tobacco control in Africa, which was acknowledged by JTI’s president and CEO: ‘This significant increase in our ownership of NTE shares reaffirms Ethiopia as an increasingly important place to do business in Africa’.^56^ Also, when announcing the purchase of a significant share in NTE, JTI claimed that ‘Ethiopia will be an essential expansion of our geographic footprint in emerging markets’, pointing to the country’s ‘double-digit economic development’ and the company’s anticipation that volumes would continue growing.^57^

The signed MOU between the Customs Commission and JTI/NTE to fight illicit trade gave NTE an opportunity to aggressively promote its products in the eastern part of the country, where illegal trade is assumed to be higher, using a display frame with a sign listing NTE products as part of putative efforts to fight illicit products.^38^ However, an independent study estimated that the illicit market share of cigarettes was 19% in 2018, less than half the TI’s estimate.^58^ Similarly, the Global Adult Tobacco Survey Ethiopia 2016 indicated that 87% of respondents who purchased cigarettes bought Nyala, which is manufactured by NTE.^59^ Similarly, the TI has used exaggerated estimates of illicit trade to offset tax hikes in other countries such as South Africa, Vietnam and Georgia.^60^,^62^ Furthermore, data on cigarette prices in Ethiopia show that, despite price rises in both legal and illicit products following the excise tax modification in 2020, the price of legal products remains lower than the price of illicit cigarettes.^63^ This implies that factors other than tobacco tax drive the illicit tobacco trade in Ethiopia.

Despite a robust tobacco control legal framework in Ethiopia, our findings demonstrate challenges persist in implementation. Poor enforcement of laws and TI interference through a range of CSR initiatives undermine the impact of Ethiopia’s new tobacco control policies. Furthermore, according to the Global Tobacco Industry Index, TI interference in Ethiopia increased from 2020 to 2021.^12 13^ Our study revealed that the TI still uses CSR activities to gain influence, meddle in life-saving health policies and secure preferential treatment from government organisations.^64^ The TI uses CSR to promote voluntary measures to control tobacco, create an illusion of being a ‘changed’ company and establish partnerships with health interests.^65^,^67^ Likewise, the industry uses corporate donations and strategic stakeholder interactions beyond those that are necessary to regulate the industry to establish and maintain close relationships with non-health government bodies, such as through the MOU with the Customs Commission (table 1). Crosbie *et al*^68^ reported over 100 ‘illicit tobacco trade’-related MOUs that were signed and publicised on TI websites, demonstrating this is a common tactic of the TI internationally.^68^ This may be related to the fact that non-health government bodies have low awareness of TI interference strategies and the laws that prohibit interaction with the TI. Similarly, recent studies conducted in Ethiopia suggest low awareness among tobacco control stakeholders with greater awareness of Article 5.3 WHO FCTC among the health sector but not among non-health government sectors.^15 16^ In addition, NTE uses other non-governmental bodies, such as the Japanese Embassy in Ethiopia and private organisations (ie, Mehrteab Leul & Associates Law Office) as avenues to interfere in tobacco control efforts and exert pressure on the government. Furthermore, our findings show that the TI interferes through working with other government-affiliated entities such as Motor & Engineering Company of Ethiopia Limited S.C. Similarly, in India, the TI has entered the food market and supports numerous government initiatives directly or indirectly through their CSR schemes.^69^ Our findings are consistent with similar TI tactics observed in Zimbabwe. The Chinese TI that operates in that country leverages its ties with the Chinese embassy in Zimbabwe as well as engaging in CSR initiatives with other economic actors and development institutions to embed its interests and benefit from perceptions of state friendship.^70^ Regular monitoring of these types of CSR activities is needed to raise the visibility of such practices and to advocate better control of TI use of CSR.

Ethiopia’s tobacco control regulatory body, EFDA, and CSOs, such as MWECS, have attempted to counter some of the TI’s CSR activities in Ethiopia. However, there are challenges in detecting and responding to interference in a timely manner. Nonetheless, some countries have successfully combated such activities by collaborating with CSOs. In Colombia and Bolivia, for example, support from international health donors, as well as ongoing participation by local health groups, improved implementation capacity to oppose TI intervention and assure effective tobacco control implementation.^71 72^ Moreover, engaging local tobacco control advocacy groups and key policymakers is a key factor for passage of tobacco control laws, as evidenced in the passage of smoke-free laws in Mexico and Ethiopia.^73 74^ Hence, governments need more comprehensive vigilance concerning TI activities involving non-health portfolios to safeguard public health policies.

Contract agreements that allow the TI to participate in lawmaking, including commenting on draft legislation, regulation or excise tax, and participating in public hearings should be banned. Hence, Ethiopia needs to learn from the experience in other countries and rescind the MOU signed between JTI and the Customs Commission in compliance with WHO FCTC Article 5.3.^75^ It is well established that the TI has exploited various voluntary agreements with the government to protect its profit while impeding the implementation of health policy in both high-income and low-income countries. Trade agreements are a notable example that give the TI an unwarranted opportunity to advocate its interests without public scrutiny.^76^ All tobacco control and public health organisations, both within and beyond Ethiopia’s health sector, should collaborate to advocate for protecting public health from the TI. The tobacco control community, particularly CSOs should be involved in promoting measures to protect public health from commercial actors. Moreover, our findings highlight the need for greater enforcement of Proclamation No. 1112/2019 to protect public health policymaking from industry involvement. Other actions that have been recommended include developing a code of conduct for all government sectors and creating a publicly accessible repository for information about the TI’s activities in Ethiopia.^15^

### Study strengths and limitations

Despite our extensive search of publicly available data, our findings may not have identified all CSR activities since the passing of Ethiopia’s strict tobacco control laws. This is due to the hidden or covert nature of TI interaction with government and other influential stakeholders. We traced publicly available data including government records to obtain information about CSR activities and other TI interference activities through targeted searches of likely data sources and information requests since there is no existing comprehensive repository to draw on. Despite these limitations, the findings provide insightful information about CSR activities in Ethiopia using available data from multiple sources. A strength of our study is the use of official letters in hard copies and documents exchanged between the government and the TI that were supplied by government departments on request.

## Conclusion

Our study demonstrates that TI interference in tobacco control efforts in Ethiopia through CSR activities continues despite laws that prohibit these activities. Our study also highlights the main TI alliances and entities that work to benefit the industry. These alliances included non-health government offices, the Japanese embassy in Addis Ababa and private law firms. Regular TI CSR monitoring and response plans are needed to develop tailored and integrated interventions to combat this interference in health policymaking. The government, in partnership with appropriate stakeholders such as CSOs, needs to monitor and address CSR breaches of SDG 3 and the WHO FCTC. Moreover, all existing agreements with NTE should be revised to comply with the new law.

## supplementary material

10.1136/tc-2023-058079 online supplemental file 1

## Data Availability

Data are available upon reasonable request. All data relevant to the study are included in the article or uploaded as supplementary information.
