# Supplementary material for: Tobacco industry corporate social responsibility activities and other interference after ratification of a strong tobacco law in Ethiopia
Source: Tob Control. 2023 Sep 15;33(6):e058079. doi: 10.1136/tc-2023-058079 (PMC11503152; doi:10.1136/tc-2023-058079)
Supplement: online supplemental file 1 [file tc-33-6-s001.pdf]

Supplementary Table 1: Source of information for identified CSR activities

S. N	Document/evidence source #	Source	date	description
1	Embassy of Japan and JTI reached an agreement to provide scholarships to Ethiopian students	Twitter[1]	2021/6/19	Detail of the scholarship has been presented with 13 trends on personal Twitter accounts.
2		Japan Embassy Website[2]	2021/5/21	On official website of Embassy of Japan, the ceremony of MOU signing between the embassy and JTI representative available.
3		Newspaper[3]	2021/5/21	Ethiopian Semonegna Newspaper reported the scholarship agreement
4		Ethiopian News Agency (ENA)[4]	2021/5/21	The Ethiopian news agency (ENA), had reported the Scholarship agreement
5	Provision of Water supply and Sanitation	Twitter[5]	April 15, 2021	JTI has announced the snapshot information about the provision of water and sanitation services for 3000 households via its official twitter account
6	NTE produced hand sanitiser and distributed for COVID-19 pandemic response.	Government internal communication	Between March and June 2020	Lideta Sub-city regulatory officer informed about this CSR activity to the tobacco industry response and monitoring team in March 2020 and EFDA reacted and stop the production and distribution of hand sanitiser by June 2020.
7		Newspaper[6]	June 6, 2020	NTE has produced hand sanitisers and distributed them to economically disadvantaged groups in the community for free.
8	NTE provide trainings for Government staff in Dire Dawa, Ethiopia	Telegram[7]		Email exchange between NTE and government office over the scheduled training and list of participated was captured by member of TIMR team and the information posted in TIMR team official Telegram channel
9		Website[8]	May 25-June 26	Regarding the number of COVID 19 case per sub-city of

				Addis Ababa reported by Daniel Kassahun
10	Participation in Green Legacy	Televised Press Conference[9]	July 26, 2019	Ethiopian Television had reported the press conference organised by Mehrteab Leul and Law firms that its employees and corporate clients including NTE to plant over 2, 500 seedlings at Adwa Park in Addis Ababa, on Monday, July 29, 2019
11		Newspaper[10]	July 26, 2019	Ethiopian Monitor, a newspaper, reported the participation of NTE in green legacy by planting 2000 seedlings as individual company and 2500 seedings as part of MLA corporate clients along other sever companies.
12		Website[11]	July 29. 2019	MOENCO reported the participation of 8 companies including NTE in planting of seedlings on July 29, 2019 green legacy day.
13	NTE contributions to the 'Tobacco Production Practice of Smallholders' on tobacco farms	Published article[12]	2020	Researcher from NTE published an article on contribution of NTE to tobacco grower farmers proximate to NTE farms
14	Tobacco Illicit Trade and excise tax	News Paper[13]	2020	Higher government officials from Ethiopia's Ministry of Finance used NTE findings on illicit trade to decide not to raise tobacco taxes.
15	Contract agreement between NTE(JTI) and government of Ethiopia and NTE uses this advantage to promote its tobacco products	Government internal document	December 19, 2017	This agreement provides enormous advantage for tobacco industry to participate in law making process, signing an agreement to fight contrabands and so on.
16		Newspaper[14]	September 16, 2019	NTE signed MOU with Customs Commission to control Illicit tobacco trade
17		Newspaper[15]	June 27, 2020	NTE bleach proclamation 1112/2019 by participating in TAPS

18	Federal Administrative Procedure Proclamation No. 1183/2020 and EFDA response to NTE request on Tobacco control directive adaptation process	Government publicly available Proclamation[16]	2020	Give detail procedures how a directive or regulation drafted, approved and stakeholder participation.
19		Government internal document	October 2020	EFDA provided a response to NTE request to get English version of Tobacco Control Directive No. 722/2021
20	Counter measure to stop production and distribution of Sanitiser by NTE	Government internal document	June, 2020	The government internal document reported that TIMRT and EFDA had successfully halted the production and distribution of COVID-19 prevention material by NTE.
21		Nwespaper[6]	June 6, 2020	MWECS exposed the NTE participation in COVID-19 response CSR active by saying this "NTE is using the current COVID-19 crisis as an opportunity to regain its image and acceptability among the government and the general public."
22	MWECS exposed NTE about breaching of TAPS law	Newspaper[15]	June 27, 2020	Accusation hits NTE for breaching ban on tobacco advertising. NTE used MOU agreement with Customs Commission to promote its tobacco products.
23	Government Tobacco Cottrol bills	Publicly Available Percolation 112/2019	January 2019	EFDA percolation that totally ban tobacco industry interferences
24		Publicly available Proclamation 1186	February 23020	Excise tax percolation that se a mixed excise tax type and recommend a periodic increase of tax rate and adjust to inflation rate.
25	FCTC implementation Guideline	Publicly available Article 5.3 implementation Guideline[17]	2013	Guidelines for implementation of Article 5.3 of the WHO Framework Convention on Tobacco Control on the protection of public health policies with respect to tobacco control from commercialand

				other vested interests of the tobacco industry
26	Government withdraws from NTE and JTI become major shareholder	Newspaper	July 18, 2016	Tobaccoreporter reported that JTI claimed that “Ethiopia will be an important expansion of our geographic footprint in emerging markets. As the largest shareholder, we expect to be able to exert significant influence over the direction of the company. The country is currently experiencing double-digit economic growth, with industry volume also expected to continue to increase.”
27		Newspaper	December 23, 2017	The Reporter reported that government withdraws from tobacco business and the JTI representative said that “This significant increase in our ownership of NTE shares reaffirms our strong belief in the company and Ethiopia as an increasingly important place to do business in Africa,”
28	Civil Society Letter to response to scholarship agreement	Internal Document	December 25, 2021	The civil society and TIMRT wrote a letter to stop NTE agreement with Embassy of Japan in Addis Ababa to stop the CSR
29	Recognition of NTE as a loyal taxpayer	Government internal document	2020	EFDA and CSOs advocate to stop awarding of NTE as best taxpayer, as a result the ministry of revenue cancelled in the list since 2020.
30	Ethiopian Tobacco Interference Index	Ethiopian Index 2020	2020[18]	Global Center for Good Governance on Tobacco Control reports about TI interference
31		Ethiopian Index 2021	2021[19]	

List of References

1. Tandon VK. Embassy of Japan and JTI reached an agreement to provide scholarships to Ethiopian students. In: @VijaiKumarTand4, editor. The Embassy of Japan in Ethiopia and Japan obacco International (JTI),signed a memorandum of understanding (MOU) on 21st May 2021 2021.

2. Ethiopia EoJi. Signing Ceremony of MoU between the Embassy of Japan and JTI (Japan Tobacco International) on a framework of cooperation to provide scholarship to Ethiopian student Addis Ababa,

Ethiopia: Embassy of Japan in Ethiopia; 2021 [Available from: https://web.archive.org/web/20230604204937/https://www.et.emb-japan.go.jp/itpr_ja/11_000001_00625.html.
3. Semonegna. Embassy of Japan and JTI reached an agreement to provide scholarships to Ethiopian students: Ethiopian Semonegna; 2021 [Available from: https://web.archive.org/web/20230604204748/https://semonegna.com/embassy-of-japan-and-jti-agreed-to-provide-scholarships-to-ethiopian-students/.
4. ENA. JTI, Japan Embassy Collaborate to Provide Scholarship for Disadvantaged Ethiopian Students Addis Ababa: Ethiopian News Agency (ENA); 2021 [updated 5/21/2021. Available from: https://web.archive.org/web/20230604203913/https://www.ena.et/web/eng/w/en_24605.
5. JTI. Last year in #Ethiopia, we initiated a program in which 3,000 households will be able to benefit from access to tap water points and new toilet facilities. Last year in #Ethiopia, we initiated a program in which 3,000 households will be able to benefit from access to tap water points and new toilet facilities. 6:37 AM ed2021.
6. Getachew S. Upping the ante against the silent killer. The Reporter. 2020 June 6, 2020.
7. Team T. List of participants from government office who are selected to attend a training organized by tobacco industry. 1:23 pm ed: Telegram; 2021.
8. Kassahun D. A story map of COVID19 cases in Addis Ababa 2020 [Available from: https://storymaps.arcgis.com/stories/a7db254fbe1543a0b15164c84bfc0551.
9. Weyna A. Seedlings Planting. Ethiopia: Ethiopian Television July 26, 2019. p. 12:00 -1: pm.
10. Monitor E. Diplomats to Partake in Tree Planting Campaign Monday. Ethiopian Monitor. 2019 July 26, 2019.
11. MOENCO. GO Green Ethiopia Addis Ababa, Ethiopia: Motor & Engineering Company of Ethiopia Limited S.C. (MOENCO), ; 2019 [Available from: https://moencoethiopia.com/blog/go-green-ethiopia/.
12. Abebe D, Tadesse M. Tobacco Production Practice of Smallholder in Bilate, Wolaita and Hawassa tobacco farms, Ethiopia. 2020.
13. G/Kristos M. Successive Tax Increase in Store for Tobacco Products. Ethiopian Monitor. 2020.
14. Astatike D. Illegal tobacco trade affects monopoly. Capital. 2019 September 16, 2019.
15. Anberbir Y. Accusation hits NTE for breaching ban on tobacco advertising. The Reporter. 2020 June 27, 2020.
16. Proclamation No. 1183/2020: A Proclamation to Provide for Federal Administrative Procedure, 1183 (2020).
17. WHO. WHO Framework Convention on Tobacco Control: guidelines for implementation Article 5.3. Geneva, Switzerland WHO; 2013.
18. GGTC. Ethiopia 2020 Tobacco Industry Index. Geneva, Swizerland: Global Center for Good Governance in Tobacco Control; 2020. Contract No.: 2/16/2023.
19. GGTC. Global Tobacco Industry Interference Index 2021: Ethiopia- country summary 2021. Geneva, Switzerland Global Center for Good Governance in Tobacco Control; 2021.
